# Vitamin D and Diabetic Kidney Disease

**DOI:** 10.3390/ijms24043751

**Published:** 2023-02-13

**Authors:** Ho-Yin Huang, Ting-Wei Lin, Zi-Xuan Hong, Lee-Moay Lim

**Affiliations:** 1 Department of Pharmacy, Kaohsiung Medical University Hospital, Kaohsiung Medical University, Kaohsiung 807, Taiwan; 2 School of Pharmacy, College of Pharmacy, Kaohsiung Medical University, Kaohsiung 807, Taiwan; 3 Graduate Institute of Clinical Medicine, College of Medicine, Kaohsiung Medical University, Kaohsiung 807, Taiwan; 4 Division of Nephrology, Department of Internal Medicine, Kaohsiung Medical University Hospital, Kaohsiung Medical University, Kaohsiung 807, Taiwan; 5 School of Medicine, College of Medicine, Kaohsiung Medical University, Kaohsiung 807, Taiwan

**Keywords:** Vitamin D, 1,25(OH)_2_D_3_, vitamin D receptor (VDR), diabetic kidney disease (DKD), diabetic nephropathy (DN)

## Abstract

Vitamin D is a hormone involved in many physiological processes. Its active form, 1,25(OH)_2_D_3_, modulates serum calcium–phosphate homeostasis and skeletal homeostasis. A growing body of evidence has demonstrated the renoprotective effects of vitamin D. Vitamin D modulates endothelial function, is associated with podocyte preservation, regulates the renin–angiotensin–aldosterone system, and has anti-inflammatory effects. Diabetic kidney disease (DKD) is a leading cause of end-stage kidney disease worldwide. There are numerous studies supporting vitamin D as a renoprotector, potentially delaying the onset of DKD. This review summarizes the findings of current research on vitamin D and its role in DKD.

## 1. Introduction

Vitamin D is essential for regulating calcium–phosphate homeostasis and promoting bone health. In addition, a growing body of evidence reveals that vitamin D is involved in a wide range of pleiotropic functions mediated by vitamin D receptors (VDR) [1]. VDR has been identified in almost all tissues, including vascular smooth muscle cells, cardiomyocytes, and endothelial cells. Vitamin D deficiency is associated with various health issues, including defects in bone mineralization, an increased risk of diabetes [2], immune defects [3], and cardiovascular diseases [4]. Several studies have demonstrated that vitamin D levels are lower in patients with diabetic kidney disease (DKD) [5,6]. In this review, we looked into the current experimental animal and human evidence regarding the role of vitamin D in DKD.

## 2. Vitamin D metabolism

**Figure 1:**
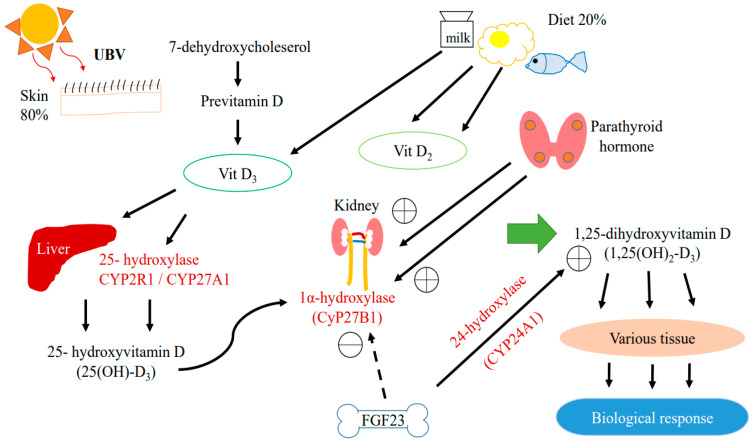
Metabolic pathway of Vitamin D.

In humans, almost 80% of vitamin D is generated in the skin in the form of vitamin D_3_ (cholecalciferol) upon exposure to ultraviolet B radiation; the remaining 20% comes from food in the forms of vitamin D_2_ (ergocalciferol) and D_3_ (Figure 1) [7]. 

Vitamin D is produced in the epidermis by photochemical transformation, which involves hydroxylating 7-dehydroxycholesterol to produce biologically active 1α,25-dihydroxyvitamin D (1,25(OH)_2_D_3_) [8,9]. This pathway involves the binding of vitamin D to vitamin D binding protein (VDBP), which is then transported to the liver and hydroxylated by 25-hydroxylase (CYP2R1) or sterol-27-hyroxylase (CYP27A1) to form 25(OH)D_3_ [10]. Both vitamin D_2_ and D_3_ can be hydroxylated by CYP2R1. Vitamin D_3_ is more commonly hydroxylated by CYP27A1 [11]. The 25(OH)D_3_ is then hydroxylated by 1α-hydroxylase either in the kidney or in peripheral tissues expressing CYP27B1 to form 1,25(OH)_2_D_3_.

1,25(OH)_2_D_3_ is the active form of vitamin D which is responsible for most of its biological activities by binding to VDR in target tissues, triggering a wide range of biological activities including the non-genomic and genetic control of signaling pathways [1]. VDR is present in various tissues in the human body, including the kidneys. VDR can be identified specifically in proximal and distal tubular epithelial cells, the parietal epithelium of the glomerulus, the juxtaglomerular apparatus, mesangial cells, collecting duct cells, and podocytes. In all cases, these VDR locations indicate that the kidneys play a crucial role in vitamin D metabolism [7,12].

Alterations of serum calcium or phosphate affect circulating levels of 1,25(OH)_2_D_3_. 1,25(OH)_2_D_3_ is regulated by parathyroid hormone (PTH) and fibroblast growth factor 23 (FGF-23). PTH activates 1α-hydroxylase when a decrease in serum calcium is detected. FGF-23 inhibits 1α-hydraxylase and activates 24-hydroxylase (CYP24A1), reducing 1,25(OH)_2_D_3_ levels [3,13]. Increased levels of CYP24A1 have been found in the kidney of animals with uremia and patients with diabetes [7,14].

## 3. Diabetic Kidney Disease

Diabetes mellitus (DM) is a metabolic disorder characterized by chronic hyperglycemia related to deficits in insulin production. Uncontrolled diabetes leads to serious damage to many organs, especially the nerves and blood vessels. DM is recognized as one of the leading causes of kidney failure by the World Health Organization [15]. According to the International Diabetes Federation (IDF), DM is estimated to be responsible for 6.7 million deaths among adults under 70 years of age, not including the mortality risk associated with COVID-19 [16]. DKD was previously known as diabetic nephropathy (DN) and is defined as elevated urine albumin excretion, decreased glomerular filtration rate (GFR), or both [17]. The clinical phase of DKD is generally divided into five stages in most guidelines characterized initially by GFR and albuminuria based on the classification and staging of chronic kidney disease (CKD) [18,19]. Morphologically, the thickening of the glomerular basement membrane, the expansion of the mesangium, glomerulosclerosis, and podocyte injury are typically revealed in kidney biopsies [20,21,22]. Approximately 40% of patients with diabetes develop DKD, risk progressing to end-stage kidney disease, and are at increased risk of cardiovascular diseases [23,24]. As the United States Renal Data System (USRDS) report, the age-standardized DKD incidence among adults aged ≥18 years with diagnosed diabetes differed greatly across countries and ranged from 81.7 to 363.6 per 100,000 diabetic population in 2014 [25]. Several factors may contribute to vitamin D deficiency in patients with diabetes, including a loss of VDBP due to proteinuria [26]. As a result of proximal tubular damage, filtered 25(OH)D_3_ bound to VDBP in the glomerular may be reabsorbed to a less extent, contributing to vitamin D deficiency in DKD [26]. Many attempts have been made to standardize the treatment of patients with DKD while minimizing kidney damage. The pleiotropic actions of vitamin D play an essential role in the management of DKD. Various hypotheses have been proposed that explain the mechanisms by which vitamin D reverses the progression of DKD, including that vitamin D assists in glucose handling, reduces the activation of the renin–angiotensin system (RAS), and reduces fibrosis [26].

## 4. Vitamin D Signaling Pathway in Diabetic Kidney Disease

Vitamin D is transported via the bloodstream throughout the human body by binding to VDBP [26]. VDBP has a low molecular weight (58 kDa) and can predict the bioavailability of 25(OH)D_3_ in the bloodstream [26]. Urinary VDBP concentrations are higher in individuals with damaged kidneys [27].

VDR is a nucleophilic protein belonging to the steroid/thyroid hormone receptor superfamily [28]. The binding of vitamin D to VDR activates the dimerization of the retinoid X receptor (RXR). This trimer binds to the VDR response element located in the promotor region of vitamin D-regulated genes via its DNA binding domain, leading to the modification of the gene expression, transcriptional response, and protein formation [26,28]. The VDR gene is located on chromosome 12q13 with several restriction enzyme sites [29]. Penna-Martinez et al. discovered that type I DM was associated with polymorphisms in these restriction enzymes [30]. A growing body of evidence suggests that the vitamin D–VDR–RXR complex regulates cell differentiation, antiproliferation, and immune modulation in the heart, kidneys, and immune system [1].

The third National Health and Nutrition Examination Survey (NHANES III) discovered that decreases in 25(OH)D levels were associated with an increase in the incidence of albuminuria in the general population [12]. Vitamin D deficiency becomes more severe as DKD progresses [31]. Several animal studies have observed lower 25(OH)D_3_ levels in a DKD group than in a control group, indicating that vitamin D plays a pivotal role in the development of DN [26,32]. A reduction in CYP27B1 activity occurs in kidney disease, which subsequently inhibits the production of 1,25(OH)_2_D_3_ and impairs the reabsorption of 25(OH)D [1]. A significant decrease in 1,25(OH)_2_D_3_ levels is observed when the GFR is 40 mL/min or less [33,34]. The decrease in CYP27B1 enzyme activity and 1,25(OH)_2_D_3_ levels can be explained by the increase in FGF-23 activity observed in the early stages of kidney disease [35,36].

## 5. Vitamin D and the Pathogenesis of Diabetic Kidney Disease

**Figure 2:**
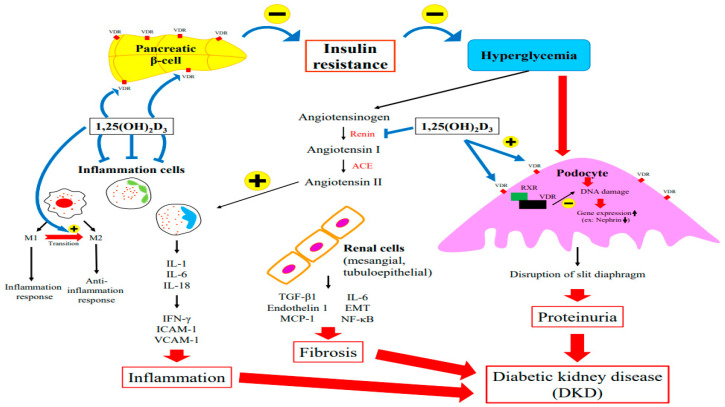
The Major Role of Vitamin D in the Pathogenesis of DKD.

Several factors contribute to DKD’s pathophysiology, including metabolic abnormalities. Hyperglycemia triggers the dysregulation of intracellular metabolism, inflammation, an increase in cell apoptosis, and tissue fibrosis [22]. The role of vitamin D in the pathogenesis of DKD is discussed in the following sections in terms of insulin resistance, podocyte injury, RAS alteration, and the inflammatory response. Figure 2 summarized the major role of vitamin D in the pathogenesis of DKD.

### 5.1. Insulin Resistance

Vitamin D deficiency is an independent risk factor for diabetes. Several studies have observed an association between vitamin D deficiency and the impairment of the glucose-mediated secretion of insulin in rat pancreatic β-cells [37,38]. Inversely, the glucose-mediated secretion of insulin seems to be restored after vitamin D supplementation [37,39].

The results from preclinical studies have indicated that vitamin D is a potential regulator of insulin secretion and Ca^2+^ influx and modulates pancreatic β-cell survival [40]. Both VDR and CYP27B1 are expressed in pancreatic β-cells. Through the binding of vitamin D to VDR, vitamin D exerts direct action toward pancreatic β-cells [41]. Several experimental studies showed that insulin synthesis decreased after glucose loading in mice without functional VDR [40,42]. In pancreatic β-cells, the VDR response element was identified in the promoter of the insulin gene, suggesting that calcitriol directly stimulates insulin release [43,44].

Both animal and clinical studies have documented an inverse relationship between low vitamin D levels and the risk of DKD [45,46]. Karnchanasorn et al. discovered that serum 25(OH)D levels are positively associated with β-cell function and insulin sensitivity [47]. An intervention study involving patients receiving hemodialysis demonstrated that 1,25(OH)_2_D_3_ administration improved glucose utilization by increasing insulin production and sensitivity [48]. Chertow et al. and Kadowaki et al. demonstrated that in rats, vitamin D deficiency was associated with impairment of insulin secretion from pancreatic β-cells [38,49]. Supplementation with vitamin D or its active derivative has been shown to improve insulin secretion [50,51].

### 5.2. Podocyte Injury

Podocytes form the outermost layer of the glomerular filtration barrier. Nephrin, podocin, and podocalyxin are proteins that constitute the slit diaphragm. Nephrin regulates the podocyte intracellular signaling pathway and plays a renoprotective role [52]. A loss of slit diaphragm integrity will lead to the appearance of proteins and other large molecules in urine and cause further damage to the kidney filtration structure. As a result of DKD, nephrin, podocin, and podocalyxin expression is reduced in podocytes while the urinary secretion of nephrin, podocin, and podocalyxin is elevated, which is consistent with proteinuria [53]. Thus, podocyte injury is one of the major causes of proteinuria and glomerulosclerosis.

Vitamin D exerts pharmacological effects via VDR on podocytes, forming a heterodimer with RXR to regulate gene expression and mediate biological activities [22,26]. VDR signaling protects podocytes from hyperglycemia-induced apoptosis and prevents DN [54]. Nakhoul et al. discovered that paricalcitol treatment, a modified form of active vitamin D and VDR agonist, was associated with the upregulation of VDR expression and a decrease in fibrosis markers such as fibronectin in their diabetic mouse model [55]. Trohatou et al. demonstrated that treatment with vitamin D_3_ and its analog ameliorated podocyte injury through the restoration of the nephrin signaling pathway [52]. Furthermore, in the same study, paricalcitol supplementation stimulated VDR expression in podocytes, induced co-localization between VDR and RXR in the nucleus, and alleviated high glucose-mediated nephrin downregulation [52]. Recently, Shi et al. revealed that active vitamin D_3_ can reverse autophagy deficiencies in the podocytes of diabetic kidneys by maintaining ATG16L1 expression and autophagy activity [56]. By modulating vitamin D_3_/VDR signaling and the downstream regulation of ATG16L1 expression, autophagy can protect podocytes from DKD-associated damage [56].

### 5.3. Suppression of the Renin–Angiotensin System

A well-known pathogenic mechanism of DN is the induction of hyperglycemia and oxidative stress through the activation of the RAS. RAS activation increases the level of angiotensin II (ANGII), which stimulates renal transforming growth factor-beta 1 (TGF-β1) production in the mesangium and epithelial tubular cells and stimulates the production of other cytokines and growth factors in renal cells, such as endothelin-1; monocyte chemoattractant protein-1 (MCP-1); interleukin-6 (IL-6); regulated upon activation, normal T cell expressed and presumably secreted (RANTES); and osteopontin. TGF-β is an important fibrogenic cytokine in the development of kidney fibrosis [57]. Elevated ANGII levels, observed in injured kidneys, cause renal inflammation and cortical damage, increase glomerular capillary pressure and permeability, intensify proteinuria, and alter renal hemodynamics [58]. In addition, ANGII is involved in the initiation of epithelial–mesenchymal transition (EMT) and renal interstitial fibrosis [59]. TGF-β activates interstitial fibroblasts and induces tubular EMT [60]. In a study by Chen et al., hyperglycemia-induced oxidative stress activated the RAS, induced EMT, and contributed to kidney fibrosis in an experimental diabetes model [61]. Vitamin D as a strong negative regulator of the RAS and suppression of renin biosynthesis has been observed in various models of kidney diseases. Eltablawy et al. observed significant inhibition of the RAS in diabetic rats receiving vitamin D supplementation [62]. Combination treatment with losartan and paricalcitrol resulted in the reversal of the aforementioned effects, reestablished the glomerular filtration barrier structure, and reduced glomerulosclerosis in diabetic mice. A study by Riera et al. demonstrated that paricalcitol, a synthetic analog of vitamin D, inhibits angiotensin-converting enzyme (ACE) 2 activity in non-obese diabetic mice and provides protection against DKD [63]. In cell cultures, 1,25(OH)_2_D suppressed renin gene transcription by a VDR-dependent mechanism [60]. In a hyperglycemic environment, 1,25(OH)_2_D also suppresses the activation of the RAS and TGF-β, abrogating tubulointerstitial fibrosis [64].

### 5.4. Inflammatory Response

Inflammation is a common feature of diabetes and can lead to DKD [65]. An inflammatory response exacerbates insulin resistance and intensifies hyperglycemia, which aggravates the long-term complications of diabetes [21,66]. A number of factors have been implicated in the pathogenesis of DKD, including the infiltration of leukocytes, monocytes, and macrophages into the kidneys [65]. Studies have supported the role of inflammatory cytokines such as IL-1, IL-6, and IL-18 in the development of DKD [67,68,69]. An increase in the levels of these molecules is associated with microvascular complications such as nephropathy [70].

IL-1 is associated with increased permeability of the endothelium of the vascular system [71]. Milas et al. investigated inflammation in early-stage DKD and discovered that urinary and plasma IL-1 levels in patients with type II diabetes are associated with podocyte and proximal tubular epithelial cell injury markers [72]. The upregulation of IL-1 in many types of kidney cells has been observed in several animal models of DKD [70,73]. IL-6 facilitates the neutrophil infiltration of the tubule interstitium; influences extracellular matrix dynamics; and promotes kidney hypertrophy, the thickening of the glomerular basement membrane, podocyte hypertrophy, and cell cycle arrest, which are factors associated with albuminuria [71]. The upregulation of IL-6 levels in patients with DKD was identified by Suzuki et al. decades ago [68]. They discovered that the mRNA expression of IL-6 in renal glomerular, epithelial, and mesangial cells was positively correlated with the severity of mesangial expansion [68]. By reducing IL-6 secretion, 1,25(OH)_2_D_3_ exerts a protective effect in DN [26]. The immunomodulatory activities of vitamin D were demonstrated by Lucisano et al. Acute supplementation with paricalcitol significantly reduced IL-17, IL-6, IL-1β, tumor necrosis factor α (TNF-α), and interferon γ (IFN-γ) in a CKD cohort [74].

IL-18 is a member of the IL-1 superfamily that stimulates the release of IFN-γ and other cytokines and modulates innate and adaptive immune cells [71]. IL-18 also stimulates the expression of intracellular adhesion molecule 1 (ICAM-1) and the production of other inflammatory cytokines in mesangial cells and is responsible for endothelial apoptosis [65,71,75]. A direct correlation between IL-18 and increased urinary albumin excretion has been confirmed. Some researchers consider IL-18 to be an early indicator of DKD [65,75]. Miyauchi et al. found that TGF-β-mediated MAPK pathway activation-induced renal tubular IL-18 expression, which was increased in patients with type 2 DKD [76]. High serum IL-18 levels have been noted in patients with macroalbuminuria, suggesting a role in the development of microvascular kidney complications [71]. Vitamin D induces CD4+ and CD25+ regulatory lymphocytes that inhibit inflammation and the effects of TNF-α, ICAM, and VCAM-1 [26,77,78].

The activation of macrophages plays a crucial role in DN [79]. Macrophages release inflammatory mediators that contribute to kidney fibrosis and the immune response [21]. The cell function of a macrophage depends on its phenotype: M1 macrophages promote tissue inflammation and tissue damage, whereas M2 macrophages are anti-inflammatory and promote tissue repair [80]. Zhang et al. demonstrated that active vitamin D_3_ promotes M1 to M2 phenotype macrophage transition, which inhibits podocyte injury and glomerular dysfunction, thereby protecting kidney function [81]. Zhang et al. also demonstrated the renoprotective effects of active vitamin D_3_ in DKD by showing that active vitamin D_3_ reduced the expression of triggering receptor expressed on myeloid cells 1 (TREM-1) and inhibited the transition of macrophages to the M1 phenotype [80]. Korf et al. demonstrated that the VDR-mediated immune signaling pathway reduces the macrophage inflammatory response and suppresses T-cell activation pathways through an IL-10-dependent mechanism [82].

The expression of proinflammatory cytokines, chemokines, and cell adhesion molecules is increased in the serum and urine of patients with DKD [12]. Transcription factor NF-κB, which regulates a variety of genes, cell adhesion molecules, chemokines, and cytokines, is a crucial inflammatory stimulus in DKD [83]. NF-κB is a key regulator in several pathways, such as the activation of the RAS, advanced glycation end-product accumulation, and nicotinamide adenine dinucleotide phosphate hydrogen-dependent oxidative stress [84,85]. NF-κB was shown to inhibit the inflammatory process, thereby alleviating the progression of kidney damage in a diabetic animal model [86]. In an experimental study on DN, Sanchez-Nino et al. demonstrated that VDR activation has local renal anti-inflammatory effects. VDR activation inhibits NF-κB activation in tubular and mesangial cells [87]. Using in vivo and in vitro experiments, Liu et al. revealed that high levels of 1,25(OH)_2_D_3_ protect against tubulointerstitial fibrosis by downregulating the expression of TLR4-MyD88-NF-kB [88].

## 6. Role of Vitamin D in Management of Diabetic Kidney Disease

Patients with diabetes and low serum levels of vitamin D are at an increased risk of DKD and the subsequent deterioration of renal function [45,46,89,90,91,92]. A new strategy for treating DKD involves supplementation with vitamin D in addition to conventional glycemic control and RAS blockade [93]. However, whether vitamin D supplementation benefits patients with DKD with suboptimal vitamin D levels is controversial (Table 1). A previous study revealed that RAS inhibitors, particularly ACE inhibitors and ANGII receptor blockers, in combination with vitamin D supplementation, were associated with an increased reduction in albuminuria. A recent systematic review and meta-analysis assessed the effects of different types of vitamin D on 1464 patients with DKD [66]. Calcitriol, alfacalcidol, and vitamin D_3_ reduced urinary protein excretion and levels of key inflammatory markers, including high-sensitivity C-reactive protein (hs-CRP), TNF-α, and IL-6, but had no effects on serum creatinine, eGFR, or glycemic control.

### 6.1. Vitamin D Status in Diabetic Kidney Disease

Patients with DKD are more likely to be vitamin D deficient than those without DKD [6,108,109,110,111,112,113,114,115]. A cross-sectional study involving 300 patients with diabetes found that CKD was significantly associated with vitamin D deficiency (*p* = 0.043). Patients with CKD were 1.7 times more likely to be vitamin D deficient than those without CKD [6]. In another study involving 448 patients with DM, serum 25(OH)D levels were significantly lower in patients with DN than in those without DN [8.5 (interquartile range 6.8–11.3) vs. 13.9 (interquartile range 11.2–18.2) ng/mL, *p* < 0.0001]. Moreover, an optimal 25(OH)D cutoff of 10.5 ng/mL (a 6.559-fold increased risk of DN) was identified as an indicator for the diagnosis of DN [108]. In 161 patients with type II DM with biopsy-proven DN, levels of 25(OH)D of less than 5 ng/mL were associated with worse renal function, pathological injury, and poor renal prognoses [92]. In a retrospective study involving 182 patients with type II DM and CKD (stages 1–4), the risk of CKD progression was significantly greater among patients with the lowest tertile of serum 25(OH)D levels than among those with the highest tertile of serum 25(OH)D levels (*p* = 0.03). Lower baseline and time-weighted average serum 25(OH)D levels were associated with an increased risk of CKD progression, which suggests that the long-term maintenance of optimal vitamin D levels from early in life might be associated with a reduced risk of CKD in patients with type II DM [113]. By contrast, no significant relationship between baseline serum levels of vitamin D and urine albumin to creatinine ratio (UACR) was observed in patients with DN compared with those without DN [102,116].

A retrospective observational study involving 240 patients with CKD and 60 patients without CKD investigated the association of 25(OH)D levels with renal function at different CKD stages. Patients were sequentially grouped according to the CKD stage. Serum 25(OH)D levels were significantly lower in patients with CKD than in those without CKD (*p* < 0.05) and were positively correlated with CKD stage (CKD 5: 7.74 ± 2.90, CKD 4: 8.44 ± 2.53, CKD 3: 10.31 ± 3.36, CKD 1–2: 12.23 ± 4.07, vs. control: 29.43 ± 10.15 ng/mL) [111]. In 502 patients with type II DM grouped according to CKD stage, significantly lower levels of 25(OH)D_3_ were observed in patients who were microalbuminuric (UACR 30–300 µg/mg) or macroalbuminuric (UACR > 300 µg/mg) than in patients who were normoalbuminuric (UACR < 30 µg/mg; *p* < 0.01). A significant positive correlation between 25(OH)D levels and eGFR was observed (r = 2.785, *p* < 0.001) [109]. Ray S et al. in their cross-sectional study evaluated vitamin D levels in 72 patients with diabetes with newly diagnosed stage 4 and 5 CKD [112]. The Vitamin D level in patients with stage 4 CKD was 19.15 (interquartile range 13.6–23.4) ng/mL and that in patients with stage 5 CKD was 10.95 (interquartile range 9.3–16.4) ng/mL (*p* = 0.006). A significant negative correlation between UACR and 25(OH)D levels was also observed (*p* = 0.002) [112]. A nonlinear relationship between 25(OH)D and UACR was found in a more recent retrospective study that enrolled 549 participants with type II DM. Serum 25(OH)D levels were negatively correlated with UACR when 25(OH)D levels were less than 67 nmol/L [114].

Two studies have investigated the association of vitamin D levels with all-cause mortality [110,115]. In a study involving 12,763 patients with diabetes with stage 3 or 4 CKD, the patients were divided into three groups according to vitamin D level: <15, 15–29, and 30 ng/mL. 25(OH)D levels of <15 ng/mL were associated with a 34% higher risk of all-cause mortality [110]. Another recent cross-sectional study that involved a total of 1202 patients with DKD categorized into quartiles based on 25(OH)D levels (<25.0, 25.0–49.9, 50.0–74.9, and ≥75 nmol/L) demonstrated that higher 25(OH)D levels were significantly correlated with a lower risk of mortality (*p* for trend = 0.003). For per one-unit increment in natural log-transformed 25(OH)D level, an 18% lower risk of all-cause mortality was observed [115]. These findings indicate that maintaining adequate vitamin D levels has potential advantages in the primary prevention of mortality among individuals with DKD.

### 6.2. Treatment with Vitamin D and its Analogs for Diabetic Kidney Disease

Several well-designed observational and interventional studies have reported the recovery of renal function after vitamin D therapy in patients with DKD [94,96,98,99,100]. Different forms of vitamin D medication are listed in Table 2. An open-label randomized controlled trial (RCT) revealed that calcitriol (0.5 μg twice weekly) treatment for 16 weeks resulted in an 18.7% decrease in the urine protein to creatinine ratio in the calcitriol group compared with a 9.9% increase in the control group [94]. In a double-blind RCT, patients with DN were given 50,000 IU per month of intramuscular vitamin D_3_ or a placebo for 6 months; the patients in the treatment group exhibited a significant reduction in UACR (51.8 mg/g) and an increase in GFR (7.0 mL/min) compared with the placebo group [98]. A prospective study observed that urinary albumin excretion decreased significantly (from 127.05 ± 84.79 to 104.81 ± 74.05 μg/mg) in patients with type I DM with microalbuminuria with vitamin D deficiency after oral administration of 0.25 μg calcitriol per day for 6 months [96]. Lower levels of urinary protein (*p* = 0.006) were observed in an oral calcitriol 50,000 IU weekly treatment group than in a placebo group, but no significant differences in serum creatinine and GFR were observed at 8 weeks between the groups [99]. A few studies have shown no statistically significant benefit of vitamin D therapy on renal function [95,97]. A cross-sectional study observed a non-significant reduction in urinary albumin excretion in patients with type II DM with albuminuria with vitamin D deficiency receiving 0.5 μg of calcitriol daily for 8 weeks (*p* = 0.22) [95].

Inflammation plays a pivotal role in the progression of DN. Patients with vitamin D deficiency exhibited higher inflammatory markers compared with those with vitamin D sufficiency [96]. Vitamin D attenuates DN-induced proteinuria by suppressing the secretion of inflammatory cytokines. Treatment with 0.25 μg of oral calcitriol daily for 6 months significantly reduced levels of serum and urinary inflammatory markers, including MCP-1, TGF-β, IL-6, and TNF-α in patients with type I DM with microalbuminuria [96]. A meta-analysis involving 284 patients with DKD from three RCTs observed that levels of IL-6 and TNF-α decreased by 0.73 mg/L and 56.79 mg/L, respectively, after supplementation with calcitriol (*p* < 0.00001) [66]. Seven RCTs involving 534 patients with DKD observed a significant decrease in hs-CRP after supplementation with vitamin D (*p* < 0.00001) [66]. However, a study by Thethi et al. observed no statistically significant differences in changes in the levels of inflammatory cytokines (ICAM-1, MCP-1, TNF-α, IL-6, and hs-CRP) between a treatment group receiving paricalcitol and a placebo group [97].

Whether vitamin D supplementation supports glycemic control is unclear. No significant changes in fasting blood glucose (FBG), glycated hemoglobin (HbA1c), the area under the curve of C-peptide, and daily insulin consumption were observed after treatment with 0.25 μg of calcitriol daily for 6 months, suggesting that vitamin D supplementation does not alter glucose metabolism [96]. A previous study observed a significant improvement in HbA1C (*p* = 0.014) but not FBG (*p* = 0.16) in patients with DKD receiving 0.5 μg of calcitriol daily for 8 weeks [95]. A meta-analysis involving a relatively small number of studies investigating the effects of vitamin D on glycemic control in patients with DKD observed no differences in HbA1C and FBG between a vitamin D treatment group and a control group [66].

Previous reports have indicated that vitamin D treatment has a beneficial effect on risk factors for DN such as hyperlipidemia and hypertension. A cross-sectional study involving 119 outpatients with type II DM with albuminuria observed significant reductions in diastolic blood pressure (*p* = 0.004) and levels of total cholesterol (TC) (*p* = 0.019) and low-density lipoprotein (LDL) (*p* = 0.04) and an increase in levels of high-density lipoprotein (*p* = 0.001) after treatment with vitamin D [95]. Significant reductions in the serum levels of triglyceride, LDL, and TC were also observed (*p* = 0.04, *p* = 0.006, and *p* = 0.02, respectively) in patients with DKD receiving 50,000 IU of oral vitamin D per week for 8 weeks [99]. Additionally, a double-blind RCT observed significant reductions in total bone mineral density and bone mineral content in patients with DN receiving 50,000 IU of intramuscular vitamin D_3_ per month for 6 months compared with a treatment group (*p* = 0.009) [100].

### 6.3. Synergistic Drug Combinations of Vitamin D and RAS Inhibitors

A novel treatment strategy for preventing DKD progression that involves a combination of VDR activation and RAS inhibition has been proposed [101,102,103,104,105,106,107]. In the VITAL study, a significant decrease (28%) in albumin excretion and the maintenance of a stable eGFR was demonstrated in patients with type II DM with albuminuria receiving RAS inhibitors and 2 µg of paricalcitol daily compared with a control group [101]. Antiproteinuric effects of paricalcitol were also observed in patients with type I DM with renal impairment [105]. Two observational studies observed a significant reduction in UACR but no change in eGFR after combination treatment with vitamin D_3_ and RAS inhibitors [102,103]. An RCT conducted by Tiryaki et al. demonstrated that supplementation with 0.25 μg of calcitriol daily for 6 months in conjunction with RAS inhibition induced significant reductions in UACR and the urine angiotensinogen/creatinine ratio compared with baseline [107]. This indicates that VDR activation might blunt albuminuria by reducing urinary angiotensinogen levels, reflecting intrarenal RAS status. The additive effects of RAS blockade and vitamin D on proteinuria control may be attributed to the reduced FGF-23 levels as a consequence of RAS inhibitor therapy, which increases the bioavailability of active 1,25(OH)_2_D_3_ [117,118]. However, no statistically significant difference in UACR was observed in patients with type II DM with vitamin D deficiency receiving 50,000 IU of vitamin D weekly for 3 months under RAS blockade [104].

### 6.4. Potential Side Effects of Vitamin D Treatment

Vitamin D therapy has not been associated with serious adverse events. Hypercalcemia and increased calcium–phosphate products are known to occur more frequently in patients treated with vitamin D in some but not all trials [119,120]. Episodes of hypercalcemia were more frequent in the paricalcitol group compared with the placebo group in the PRIMO RCT (paricalcitol: 22.6% vs. placebo: 0.9%; *p* < 0.001) [121]. The incidence of hypercalcemia was similar between the groups receiving either 1 or 2 μg paricalcitol versus placebo in the VITAL RCT (*p* > 0.99 vs. placebo and *p* = 0.62 vs. placebo, respectively) [101]. Notably, a small but significant reduction in creatinine-based measures of eGFR was observed in both the PRIMO and VITAL studies [101,121]. Although this finding could reflect a true GFR reduction with conventional RAS blockade, a possible explanation might be an effect on creatinine metabolism or inappropriate eGFR measures reported with paricalcitol or calcitriol treatment in patients with diabetes; therefore, the finding should be interpreted with caution [119,122]. In addition, side effects such as hyperkalemia and hypotension should be considered when combining vitamin D with RAS inhibitors in an attempt to further reduce residual proteinuria [123].

## 7. Conclusions

Vitamin D deficiency is recognized as a risk factor for the development of DKD. The mechanisms by which vitamin D reverses the progression of DKD, including that vitamin D assists in glucose handling, protects podocytes from apoptosis, reduces the activation of the RAS, reduces fibrosis, and exhibits anti-inflammatory effects, are manifold and still largely remain speculative. It is expected that vitamin D supplementation may be beneficial for DKD patients regarding its possible renoprotective role; however, controversial results are shown in several interventional studies. Furthermore, there are currently no recommendations about the optimal dosage and timing of vitamin D treatment in DKD. More research into the interindividual variability of vitamin D metabolism and different response to vitamin D regimens are needed in the future.

In summary, the studies reviewed here emphasize the possible roles of vitamin D beyond calcium–phosphate homeostasis modulation in DKD. Experimental studies, observational studies, and clinical trials have indicated the possible effects of vitamin D in protecting against the progression of DKD and preserving the integrity of the glomerular filtration barrier. These studies have highlighted the necessity of clinicians to be alert to vitamin D deficiency in patients with DKD and the importance of supplementation among high-risk groups.

## Figures and Tables

**Table 1:** Main findings from clinical studies of vitamin D treatment in DKD patients.

Study	Design	Population	Intervention	End point Measures	Main Results
Vitamin D and its analogs					
Krairittichai et al. [94] (2012)	Randomized controlled trial	T2DM, UPCR> 1 g/g, eGFR> 15 mL/min/1.73 m^2^ (n = 91)	Oral calcitriol 0.5 μg twice weekly for 16 weeks (n = 46)	UPCR, eGFR	Percent changes in UPCR (−18.7% vs. +9.9%, *p* < 0.01), a 30% or more decrement in proteinuria (43.5% vs. 11.1%, *p* < 0.01), mean eGFR (35.5 vs. 36.9 mL/min/1.73 m^2^, *p* = 0.83) in treatment group vs. placebo.
Bonakdaran et al. [95] (2012)	Cross-sectional study	T2DM, albuminuria (n = 119)	Oral calcitriol 0.5 μg daily for 8 weeks (n = 43)	Albumin excretion rate, FBS, HbA1C, hs-CRP, lipid profile	Albumin excretion rate (73.1 to 57.1, *p* = 0.22) in vitamin D insufficiency/deficiency group.
Mao et al. [96] (2014)	Prospective observational study	T1DM, microalbuminuria (n = 31)	Oral calcitriol 0.25 μg daily for 6 months (n = 24)	Urinary albumin excretion, urine MCP-1, urine TGF-β, TNF-α, IL-6, C-peptide, HbA1C, serum calcium, phosphorus, PTH, 25(OH)D levels, CRP	Urinary albumin excretion (127.05 to 104.81 μg/mg, *p* < 0.05), urine MCP-1/creatinine (99.38 to 89.57 ng/mmol, *p* < 0.05), urine TGF-β/creatinine (79 to 72.33 ng/mmol, *p* < 0.05), TNF-α (57.7 to 47.09 pg/mL, *p* < 0.05), IL-6 (44.04 to 39.88 pg/mL, *p* < 0.05) in vitamin D insufficiency/deficiency group.
Thethi et al. [97] (2015)	Randomized controlled trial	T2DM, eGFR 15–59 mL/min/1.73 m^2^ (n = 60)	Oral paricalcitol 1 μg daily for 3 months (n = 27)	IL-6, hs-CRP, TNF-α, MCP-1, ICAM-1	No significant differences in outcomes between the paricalcitol and placebo groups.
Liyanage et al. [98] (2018)	Randomized controlled trial	DN, UACR > 30 mg/g, GFR > 30 mL/min (n = 82)	IM vitamin D_3_ 50,000 IU monthly for 6 months (n = 41)	Plasma rennin, UACR, GFR, serum creatinine	Plasma renin (8.83 vs. 14.19 pg/mL, *p* = 0.006), urine albumin (117.6 vs.163.4 mg/g, *p* = 0.001), serum creatinine (0.77 vs. 0.87 mg/dL, *p* = 0.10), GFR (93.7 vs. 83.9 mL/min, *p* = 0.03) in treatment group vs. placebo.
Barzegari et al. [99] (2019)	Randomized controlled trial	DN, albuminuria ˃ 30 mg/day, GFR < 60 mL/min (n = 50)	Oral calcitriol 50,000 IU weekly for 8 weeks (n = 25)	Blood/urine parameters, oxidative/anti-oxidative markers, lipid profile	Urine protein (233.96 vs. 319.91 mg/dL, *p* = 0.006), serum creatinie (1.13 vs.1.31 μg/mL, *p* = 0.59), GFR (46.96 vs. 46.46 mL/min/1.73 m^2^, *p* = 0.81) in treatment vs. placebo.
Liyanage et al. [100] (2021)	Randomized controlled trial	DN, UACR > 30 mg/g, GFR > 30 mL/min (n = 85)	IM vitamin D3 50,000 IU monthly for 6 months (n = 42)	BMD, BMC	Total body BMD, total body BMC and BMDs of spine, femoral neck and total hip regions (+2.0%, +2.2%, +1.8%, +2.1% and +2.6%, *p* < 0.05 for all within-group differences) in treatment group.
Synergistic drug combinations					
de Zeeuw et al. [101] (2010)	Randomized controlled trial	T2DM, UACR 11–339 mg/mmol, eGFR 15–90 mL/min/1.73 m^2^, receiving stable doses of ACEIs or ARBs ≥ 3 months (n = 281)	Oral paricalcitol 1 μg daily (n = 93), or paricalcitol 2 μg daily (n = 95) for 6 months	UACR, albumin excretion rate, eGFR	UACR (−18%, *p* = 0.053), albumin excretion rate (−28%, *p* = 0.009), eGFR (−3 to −5 mL/min/1.73 m^2^, *p* = 0.001) in paricalcitol 2 μg group vs. placebo
Kim et al. [102] (2011)	Prospective observational study	T2DM, UACR > 30 mg/g, eGFR 15–90 mL/min/1.73 m^2^, receiving stable doses of ACEIs or ARBs ≥ 3 months (n = 63)	Oral vitamin D_3_ 40,000 IU weekly for 8 weeks (n = 54)	Urinary MCP-1, TGF-β_1_, UACR and eGFR at 2 and 4 months	UACR at 2 months (16.4 to 12.2 mg/mmol, *p* = 0.0011) and at 4 months (16.4 to 12 mg/mmol, *p* = 0.0201), urinary TGF-β_1_/creatinine ratio at 2 months (26.5 to 15.5 ng/mmol, *p* = 0.0095) at 4 months (26.5 to 9.5 ng/mmol, *p* < 0.0001), urinary MCP-1/creatinine ratio at 2 months (10.4 to 12.7 ng/mmol, *p* = 0.1799) at 4 months (10.4 to 11.9 ng/mmol, *p* < 0.4916), eGFR at 2 months (42 to 41 mL/min/1.73 m^2^, *p* > 0.05) at 4 months (42 to 40 mL/min/1.73 m^2^, *p* > 0.05) in treatment group.
Huang et al. [103] (2012)	Cross-sectional study	T2DM, UACR > 30 mg/g, receiving stable doses of ACEIs or ARBs ≥ 3 months (n = 46)	Oral vitamin D_3_ 800 IU daily for 6 months (n = 22)	UACR, eGFR	UACR at 2 months (97.39 to 71.65 mg/g, *p* = 0.01) and at 6 months (97.39 to 120.36 mg/g, *p* = 0.239), changes in eGFR at 6 months (*p* > 0.05) in treatment group.
Ahmadi et al. [104] (2013)	Randomized controlled trial	T2DM, UACR > 30 mg/g, receiving permanent doses of ACEIs or ARBs ≥ 3 months (n = 51)	Oral vitamin D_3_ 50,000 IU weekly for 12 weeks (n = 28)	Serum creatinine, UACR, GFR, lipid profile, HbA1C, 25(OH)D level	Serum creatinine (1.09 vs. 1.04 mg/dL, *p* = 0.251), UACR (111.49 vs. 88.43, *p* = 0.844); GFR (72.00 vs. 73.18 mL/min/1.73 m^2^, p= 0.482) in treatment group vs. placebo.
Joergensen et al. [105] (2015)	Randomized Controlled Trial	T1DM, albuminuria ≥ 300 mg/day, eGFR 15–70 mL/min/1.73 m^2^, receiving stable doses of ACEIs or ARBs (n = 48)	Oral paricalcitol 2 μg daily for 12 weeks (n = 24)	Urinary albumin excretion rate, eGFR, MR-proANP	Urinary albumin excretion rate (−18% vs. +21%, *p* = 0.03), eGFR (41 vs. 46 mL/min/1.73 m^2^, *p* = 0.0012) in treatment group vs. placebo.
Munisamy et al. [106] (2016)	Randomized controlled trial	T2DM, UACR > 30 mg/g, receiving stable doses of ACEIs or ARBs (n = 70)	Oral alfacalcidol 0.25 μg daily for 6 months (n = 34)	Microvascular endothelial function, arterial stiffness, BP, calcium, phosphate and hsCRP	CSBP (124.13 to 118.48 mmHg, *p* = 0.027); calcium (2.35 to 2.41 mmol/L, *p* = 0.002); phosphate (1.13 to 1.02 mmol/L, *p* = 0.024) in treatment group.
Tiryaki et al. [107] (2016)	Randomized controlled trial	T2DM, albuminuria, eGFR > 60 mL/min/1.73 m^2^, receiving ACEIs or ARBs ≥ 3 months (n = 98)	Oral calcitriol 0.25 μg daily for 6 months (n = 48)	Plasma PTH level, UACR, UAGT/UCre and eGFR	Plasma PTH level (*p* = 0.003), UACR (186.58 to 142.72 mg/g, *p* = 0.014), UAGT/UCre (12.96 to 8.64 mg/g, *p* = 0.012), eGFR (62.13 to 67.48, *p* = 0.09) in treatment group.

Abbreviations: T2DM, type 2 diabetes mellitus; T1DM, type 1 diabetes mellitus; DN, diabetic nephropathy; UPCR, urine protein to creatinine ratio; eGFR, estimated glomerular filtration rate; UACR, urine albumin to creatinine ratio; ACEIs, angiotensin- converting enzyme inhibitors; ARBs, angiotensin receptor blockers; IM, intramuscular; HS-CRP, high-sensitive C-reactive protein; FBS, fasting blood sugar; HbA1C, glycated hemoglobin; MCP-1, monocyte chemoattractant protein-1; ICAM-1, intercellular adhesion molecule-1; TGF-β, transforming growth factor-β; TNF-α, tumor necrosis factor-α; IL-6, interleukin 6; BMD, bone mineral density; BMC, bone mineral content; CSBP, central systolic blood pressure; MR-proANP, mid-regional pro-atrial natriuretic peptide; UAGT, urinary angiotensinogen; PTH, pararthyroid hormon.

**Table 2:** Different forms of vitamin D medication.

Generic Name	Brand Name	Dosage Forms and Common Formulations
Inactive precursors		
Ergocalciferol (Vitamin D_2_)	Calcidol, Calciferol, Drisdol	Oral capsule (50 mcg; 1250 mcg) Oral liquid (200 mcg/mL)
Cholecalciferol (Vitamin D_3_)	Carlson D, Ddrops, Decara, Delta D3, Enfamil D-Vi-Sol, Replesta, Thera-D Rapid Repletion, UpSpringbaby D, etc.	Oral capsule (2000 IU; 5000 IU; 10,000 IU; 50,000 IU) Oral tablet (400 IU; 1000 IU; 5000 IU; 10,000 IU; 50,000 IU) Oral liquid (10,000 IU/mL; 25,000 IU/mL; 50,000 IU/mL) Sublingual liquid (5000 IU/mL) Injectable solution (300,000 IU/mL; 600,000 IU/mL)
Alfacalcidol (1α-hydroxy vitamin D_3_)	One-Alpha	Oral capsule (0.5 mcg; 1 mcg) Oral drops (2 mcg/mL) Injectable solution (2 mcg/mL)
Active forms		
Calcitriol (1,25-dihydroxyvitamin D_3_)	Rocaltrol, Calcijex	Oral capsule (0.25 mcg; 0.5 mcg) Oral liquid (1 mcg/mL) Injectable solution (1 mcg/mL)
Paricalcitol	Zemplar	Injectable solution (2 mcg/mL; 5 mcg/mL) Oral capsule (1 mcg; 2 mcg; 4 mcg)
Maxacalcitol	Oxarol	Injectable solution (2.5 mcg/mL)
Doxercalciferol	Hectorol	Injectable solution (2 mcg/mL) Oral capsule (0.5 mcg; 1 mcg; 2.5 mcg)

Abbreviations: IU, international unit.

## Data Availability

Not applicable.

## References

[1] Yang S., Li A., Wang J., Liu J., Han Y., Zhang W., Li Y.C., Zhang H. (2018). Vitamin D Receptor: A Novel Therapeutic Target for Kidney Diseases. Curr. Med. Chem..

[2] Sacerdote A., Dave P., Lokshin V., Bahtiyar G. (2019). Type 2 Diabetes Mellitus, Insulin Resistance, and Vitamin D. Curr. Diab. Rep..

[3] Gil A., Plaza-Diaz J., Mesa M.D. (2018). Vitamin D: Classic and Novel Actions. Ann. Nutr. Metab..

[4] Latic N., Erben R.G. (2020). Vitamin D and Cardiovascular Disease, with Emphasis on Hypertension, Atherosclerosis, and Heart Failure. Int. J. Mol. Sci..

[5] Senyigit A. (2019). The association between 25-hydroxy vitamin D deficiency and diabetic complications in patients with type 2 diabetes mellitus. Diabetes Metab. Syndr..

[6] Jamal Shahwan M., Hassan N.A.G., Shaheen R.A. (2019). Assessment of kidney function and associated risk factors among type 2 diabetic patients. Diabetes Metab. Syndr..

[7] Galuska D., Pacal L., Kankova K. (2021). Pathophysiological Implication of Vitamin D in Diabetic Kidney Disease. Kidney Blood Press Res..

[8] Holick M.F. (2003). Vitamin D: A millenium perspective. J. Cell Biochem..

[9] Bikle D.D. (2011). Vitamin D: An ancient hormone. Exp. Dermatol..

[10] Smith J.E., Goodman D.S. (1971). The turnover and transport of vitamin D and of a polar metabolite with the properties of 25-hydroxycholecalciferol in human plasma. J. Clin. Investig..

[11] Cheng J.B., Motola D.L., Mangelsdorf D.J., Russell D.W. (2003). De-orphanization of cytochrome P450 2R1: A microsomal vitamin D 25-hydroxilase. J. Biol. Chem..

[12] Lei M., Liu Z., Guo J. (2020). The Emerging Role of Vitamin D and Vitamin D Receptor in Diabetic Nephropathy. Biomed Res. Int..

[13] Zittermann A., Trummer C., Theiler-Schwetz V., Lerchbaum E., Marz W., Pilz S. (2021). Vitamin D and Cardiovascular Disease: An Updated Narrative Review. Int. J. Mol. Sci..

[14] Tajiri M., Nakahashi O., Kagawa T., Masuda M., Ohminami H., Iwano M., Takeda E., Taketani Y., Yamamoto H. (2020). Association of increased renal Cyp24a1 gene expression with low plasma 1,25-dihydroxyvitamin D levels in rats with streptozotocin-induced diabetes. J. Clin. Biochem. Nutr..

[15] Who Health Organization (WHO) (2022). Diabetes Health Impacts.

[16] International Diabetes Federation IDF Diabetes Atlas 2021.

[17] Gheith O., Farouk N., Nampoory N., Halim M.A., Al-Otaibi T. (2016). Diabetic kidney disease: World wide difference of prevalence and risk factors. J. Nephropharmacol..

[18] Koye D.N., Shaw J.E., Reid C.M., Atkins R.C., Reutens A.T., Magliano D.J. (2017). Incidence of chronic kidney disease among people with diabetes: A systematic review of observational studies. Diabet. Med..

[19] Satirapoj B. (2010). Review on pathophysiology and treatment of diabetic kidney disease. J. Med. Assoc. Thail. Chotmaihet Thangphaet.

[20] Oshima M., Shimizu M., Yamanouchi M., Toyama T., Hara A., Furuichi K., Wada T. (2021). Trajectories of kidney function in diabetes: A clinicopathological update. Nat. Rev. Nephrol..

[21] Hu X., Liu W., Yan Y., Liu H., Huang Q., Xiao Y., Gong Z., Du J. (2019). Vitamin D protects against diabetic nephropathy: Evidence-based effectiveness and mechanism. Eur. J. Pharmacol..

[22] Fu H., Liu S., Bastacky S.I., Wang X., Tian X.J., Zhou D. (2019). Diabetic kidney diseases revisited: A new perspective for a new era. Mol. Metab..

[23] Solini A., Penno G., Bonora E., Fondelli C., Orsi E., Arosio M., Trevisan R., Vedovato M., Cignarelli M., Andreozzi F. (2012). Diverging association of reduced glomerular filtration rate and albuminuria with coronary and noncoronary events in patients with type 2 diabetes: The renal insufficiency and cardiovascular events (RIACE) Italian multicenter study. Diabetes Care.

[24] Pugliese G., Solini A., Bonora E., Fondelli C., Orsi E., Nicolucci A., Penno G., Group R.S. (2014). Chronic kidney disease in type 2 diabetes: Lessons from the Renal Insufficiency And Cardiovascular Events (RIACE) Italian Multicentre Study. Nutr. Metab. Cardiovasc. Dis..

[25] Burrows N.R., Hora I., Geiss L.S., Gregg E.W., Albright A. (2017). Incidence of End-Stage Renal Disease Attributed to Diabetes Among Persons with Diagnosed Diabetes—United States and Puerto Rico, 2000–2014. MMWR. Morb. Mortal. Wkly. Rep..

[26] Delrue C., Speeckaert R., Delanghe J.R., Speeckaert M.M. (2022). The Role of Vitamin D in Diabetic Nephropathy: A Translational Approach. Int. J. Mol. Sci..

[27] Song Z., Xiao C., Jia X., Luo C., Shi L., Xia R., Zhu J., Zhang S. (2021). Vitamin D/VDR Protects Against Diabetic Kidney Disease by Restoring Podocytes Autophagy. Diabetes Metab. Syndr. Obes..

[28] Pike J.W., Meyer M.B., Lee S.M., Onal M., Benkusky N.A. (2017). The vitamin D receptor: Contemporary genomic approaches reveal new basic and translational insights. J. Clin. Investig..

[29] Uitterlinden A.G., Fang Y., Van Meurs J.B., Pols H.A., Van Leeuwen J.P. (2004). Genetics and biology of vitamin D receptor polymorphisms. Gene.

[30] Penna-Martinez M., Badenhoop K. (2017). Inherited Variation in Vitamin D Genes and Type 1 Diabetes Predisposition. Genes.

[31] Chokhandre M.K., Mahmoud M.I., Hakami T., Jafer M., Inamdar A.S. (2015). Vitamin D & its analogues in type 2 diabetic nephropathy: A systematic review. J. Diabetes Metab. Disord..

[32] Wang H., Wang J., Qu H., Wei H., Ji B., Yang Z., Wu J., He Q., Luo Y., Liu D. (2016). In vitro and in vivo inhibition of mTOR by 1,25-dihydroxyvitamin D3 to improve early diabetic nephropathy via the DDIT4/TSC2/mTOR pathway. Endocrine.

[33] Levin A., Bakris G.L., Molitch M., Smulders M., Tian J., Williams L.A., Andress D.L. (2007). Prevalence of abnormal serum vitamin D, PTH, calcium, and phosphorus in patients with chronic kidney disease: Results of the study to evaluate early kidney disease. Kidney Int..

[34] Turner M.E., Rowsell T.S., White C.A., Kaufmann M., Norman P.A., Neville K., Petkovich M., Jones G., Adams M.A., Holden R.M. (2022). The metabolism of 1,25(OH)2D3 in clinical and experimental kidney disease. Sci. Rep..

[35] Kagi L., Bettoni C., Pastor-Arroyo E.M., Schnitzbauer U., Hernando N., Wagner C.A. (2018). Regulation of vitamin D metabolizing enzymes in murine renal and extrarenal tissues by dietary phosphate, FGF23, and 1,25(OH)2D3. PLoS ONE.

[36] Perwad F., Zhang M.Y., Tenenhouse H.S., Portale A.A. (2007). Fibroblast growth factor 23 impairs phosphorus and vitamin D metabolism in vivo and suppresses 25-hydroxyvitamin D-1alpha-hydroxylase expression in vitro. Am. J. Physiol. Renal. Physiol..

[37] Bornstedt M.E., Gjerlaugsen N., Pepaj M., Bredahl M.K.L., Thorsby P.M. (2019). Vitamin D Increases Glucose Stimulated Insulin Secretion from Insulin Producing Beta Cells (INS1E). Int. J. Endocrinol. Metab..

[38] Chertow B.S., Sivitz W.I., Baranetsky N.G., Clark S.A., Waite A., Deluca H.F. (1983). Cellular mechanisms of insulin release: The effects of vitamin D deficiency and repletion on rat insulin secretion. Endocrinology.

[39] Wimalawansa S.J. (2018). Associations of vitamin D with insulin resistance, obesity, type 2 diabetes, and metabolic syndrome. J. Steroid. Biochem. Mol. Biol..

[40] Szymczak-Pajor I., Sliwinska A. (2019). Analysis of Association between Vitamin D Deficiency and Insulin Resistance. Nutrients.

[41] Bland R., Markovic D., Hills C.E., Hughes S.V., Chan S.L., Squires P.E., Hewison M. (2004). Expression of 25-hydroxyvitamin D3-1alpha-hydroxylase in pancreatic islets. J. Steroid. Biochem. Mol. Biol..

[42] Morro M., Vila L., Franckhauser S., Mallol C., Elias G., Ferre T., Molas M., Casana E., Rodo J., Pujol A. (2020). Vitamin D Receptor Overexpression in beta-Cells Ameliorates Diabetes in Mice. Diabetes.

[43] Kjalarsdottir L., Tersey S.A., Vishwanath M., Chuang J.C., Posner B.A., Mirmira R.G., Repa J.J. (2019). 1,25-Dihydroxyvitamin D3 enhances glucose-stimulated insulin secretion in mouse and human islets: A role for transcriptional regulation of voltage-gated calcium channels by the vitamin D receptor. J. Steroid. Biochem. Mol. Biol..

[44] Altieri B., Grant W.B., Della Casa S., Orio F., Pontecorvi A., Colao A., Sarno G., Muscogiuri G. (2017). Vitamin D and pancreas: The role of sunshine vitamin in the pathogenesis of diabetes mellitus and pancreatic cancer. Crit. Rev. Food Sci. Nutr..

[45] Schottker B., Herder C., Rothenbacher D., Perna L., Muller H., Brenner H. (2013). Serum 25-hydroxyvitamin D levels and incident diabetes mellitus type 2: A competing risk analysis in a large population-based cohort of older adults. Eur. J. Epidemiol..

[46] Tsur A., Feldman B.S., Feldhammer I., Hoshen M.B., Leibowitz G., Balicer R.D. (2013). Decreased serum concentrations of 25-hydroxycholecalciferol are associated with increased risk of progression to impaired fasting glucose and diabetes. Diabetes Care.

[47] Karnchanasorn R., Ou H.Y., Chiu K.C. (2012). Plasma 25-hydroxyvitamin D levels are favorably associated with beta-cell function. Pancreas.

[48] Hosseini E.S., Kashani H.H., Nikzad H., Soleimani A., Mirzaei H., Tamadon M.R., Asemi Z. (2019). Diabetic Hemodialysis: Vitamin D Supplementation and its Related Signaling Pathways Involved in Insulin and Lipid Metabolism. Curr. Mol. Med..

[49] Kadowaki S., Norman A.W. (1984). Dietary vitamin D is essential for normal insulin secretion from the perfused rat pancreas. J. Clin. Investig..

[50] Kawahara T., Suzuki G., Mizuno S., Inazu T., Kasagi F., Kawahara C., Okada Y., Tanaka Y. (2022). Effect of active vitamin D treatment on development of type 2 diabetes: DPVD randomised controlled trial in Japanese population. BMJ.

[51] Lemieux P., Weisnagel S.J., Caron A.Z., Julien A.S., Morisset A.S., Carreau A.M., Poirier J., Tchernof A., Robitaille J., Bergeron J. (2019). Effects of 6-month vitamin D supplementation on insulin sensitivity and secretion: A randomised, placebo-controlled trial. Eur. J. Endocrinol..

[52] Trohatou O., Tsilibary E.F., Charonis A., Iatrou C., Drossopoulou G. (2017). Vitamin D3 ameliorates podocyte injury through the nephrin signalling pathway. J. Cell Mol. Med..

[53] Wang R., Yao C., Liu F. (2020). Association between Renal Podocalyxin Expression and Renal Dysfunction in Patients with Diabetic Nephropathy: A Single-Center, Retrospective Case-Control Study. Biomed. Res. Int..

[54] Deb D.K., Wang Y., Zhang Z., Nie H., Huang X., Yuan Z., Chen Y., Zhao Q., Li Y.C. (2011). Molecular mechanism underlying 1,25-dihydroxyvitamin D regulation of nephrin gene expression. J. Biol. Chem..

[55] Nakhoul N., Thawko T., Farber E., Dahan I., Tadmor H., Nakhoul R., Hanut A., Salameh G., Shagrawy I., Nakhoul F. (2020). The Therapeutic Effect of Active Vitamin D Supplementation in Preventing the Progression of Diabetic Nephropathy in a Diabetic Mouse Model. J. Diabetes Res..

[56] Shi L., Xiao C., Zhang Y., Xia Y., Zha H., Zhu J., Song Z. (2022). Vitamin D/vitamin D receptor/Atg16L1 axis maintains podocyte autophagy and survival in diabetic kidney disease. Ren Fail..

[57] Langham R.G., Kelly D.J., Gow R.M., Zhang Y., Cordonnier D.J., Pinel N., Zaoui P., Gilbert R.E. (2006). Transforming growth factor-beta in human diabetic nephropathy: Effects of ACE inhibition. Diabetes Care.

[58] Lin L., Phillips W.E., Manning R.D. (2009). Intrarenal Angiotensin ii is associated with inflammation, renal damage and dysfunction in dahl salt-sensitive hypertension. J. Am. Soc. Hypertens.

[59] Gong Q., Hou F. (2016). Silencing of angiotensin II type-1 receptor inhibits high glucose-induced epithelial-mesenchymal transition in human renal proximal tubular epithelial cells via inactivation of mTOR/p70S6K signaling pathway. Biochem. Biophys. Res. Commun..

[60] Agarwal R. (2009). Vitamin D, proteinuria, diabetic nephropathy, and progression of CKD. Clin. J. Am. Soc. Nephrol..

[61] Chen C.M., Juan S.H., Chou H.C. (2018). Hyperglycemia activates the renin-angiotensin system and induces epithelial-mesenchymal transition in streptozotocin-induced diabetic kidneys. J. Renin. Angiotensin Aldosterone Syst..

[62] Eltablawy N., Ashour H., Rashed L.A., Hamza W.M. (2018). Vitamin D protection from rat diabetic nephropathy is partly mediated through Klotho expression and renin-angiotensin inhibition. Arch. Physiol. Biochem..

[63] Riera M., Anguiano L., Clotet S., Roca-Ho H., Rebull M., Pascual J., Soler M.J. (2016). Paricalcitol modulates ACE2 shedding and renal ADAM17 in NOD mice beyond proteinuria. Am. J. Physiol. Renal. Physiol..

[64] Zhang Z., Sun L., Wang Y., Ning G., Minto A.W., Kong J., Quigg R.J., Li Y.C. (2008). Renoprotective role of the vitamin D receptor in diabetic nephropathy. Kidney Int..

[65] Samsu N. (2021). Diabetic Nephropathy: Challenges in Pathogenesis, Diagnosis, and Treatment. Biomed. Res. Int..

[66] Wang Y., Yang S., Zhou Q., Zhang H., Yi B. (2019). Effects of Vitamin D Supplementation on Renal Function, Inflammation and Glycemic Control in Patients with Diabetic Nephropathy: A Systematic Review and Meta-Analysis. Kidney Blood Press Res..

[67] Navarro J.F., Mora C. (2005). Role of inflammation in diabetic complications. Nephrol. Dial. Transplant..

[68] Suzuki D., Miyazaki M., Naka R., Koji T., Yagame M., Jinde K., Endoh M., Nomoto Y., Sakai H. (1995). In situ hybridization of interleukin 6 in diabetic nephropathy. Diabetes.

[69] Wong C.K., Ho A.W., Tong P.C., Yeung C.Y., Kong A.P., Lun S.W., Chan J.C., Lam C.W. (2007). Aberrant activation profile of cytokines and mitogen-activated protein kinases in type 2 diabetic patients with nephropathy. Clin. Exp. Immunol..

[70] Donate-Correa J., Ferri C.M., Sanchez-Quintana F., Perez-Castro A., Gonzalez-Luis A., Martin-Nunez E., Mora-Fernandez C., Navarro-Gonzalez J.F. (2020). Inflammatory Cytokines in Diabetic Kidney Disease: Pathophysiologic and Therapeutic Implications. Front. Med..

[71] Perez-Morales R.E., Del Pino M.D., Valdivielso J.M., Ortiz A., Mora-Fernandez C., Navarro-Gonzalez J.F. (2019). Inflammation in Diabetic Kidney Disease. Nephron.

[72] Milas O., Gadalean F., Vlad A., Dumitrascu V., Velciov S., Gluhovschi C., Bob F., Popescu R., Ursoniu S., Jianu D.C. (2020). Pro-inflammatory cytokines are associated with podocyte damage and proximal tubular dysfunction in the early stage of diabetic kidney disease in type 2 diabetes mellitus patients. J. Diabetes Complicat..

[73] Navarro J.F., Mora C., Muros M., Garcia J. (2006). Urinary tumour necrosis factor-alpha excretion independently correlates with clinical markers of glomerular and tubulointerstitial injury in type 2 diabetic patients. Nephrol. Dial. Transpl..

[74] Lucisano S., Arena A., Stassi G., Iannello D., Montalto G., Romeo A., Costantino G., Lupica R., Cernaro V., Santoro D. (2015). Role of Paricalcitol in Modulating the Immune Response in Patients with Renal Disease. Int. J. Endocrinol..

[75] Lin Y.C., Chang Y.H., Yang S.Y., Wu K.D., Chu T.S. (2018). Update of pathophysiology and management of diabetic kidney disease. J. Med. Assoc..

[76] Miyauchi K., Takiyama Y., Honjyo J., Tateno M., Haneda M. (2009). Upregulated IL-18 expression in type 2 diabetic subjects with nephropathy: TGF-beta1 enhanced IL-18 expression in human renal proximal tubular epithelial cells. Diabetes Res. Clin. Pract..

[77] Sirbe C., Rednic S., Grama A., Pop T.L. (2022). An Update on the Effects of Vitamin D on the Immune System and Autoimmune Diseases. Int. J. Mol. Sci..

[78] Faridvand Y., Bagherpour-Hassanlouei N., Nozari S., Nasiri N., Rajabi H., Ghaffari S., Nouri M. (2019). 1, 25-Dihydroxyvitamin D3 activates Apelin/APJ system and inhibits the production of adhesion molecules and inflammatory mediators in LPS-activated RAW264.7 cells. Pharmacol. Rep..

[79] Lee S., Huen S., Nishio H., Nishio S., Lee H.K., Choi B.S., Ruhrberg C., Cantley L.G. (2011). Distinct macrophage phenotypes contribute to kidney injury and repair. J. Am. Soc. Nephrol..

[80] Zhang X., Zhao Y., Zhu X., Guo Y., Yang Y., Jiang Y., Liu B. (2019). Active vitamin D regulates macrophage M1/M2 phenotypes via the STAT-1-TREM-1 pathway in diabetic nephropathy. J. Cell Physiol..

[81] Zhang X.L., Guo Y.F., Song Z.X., Zhou M. (2014). Vitamin D prevents podocyte injury via regulation of macrophage M1/M2 phenotype in diabetic nephropathy rats. Endocrinology.

[82] Korf H., Wenes M., Stijlemans B., Takiishi T., Robert S., Miani M., Eizirik D.L., Gysemans C., Mathieu C. (2012). 1,25-Dihydroxyvitamin D3 curtails the inflammatory and T cell stimulatory capacity of macrophages through an IL-10-dependent mechanism. Immunobiology.

[83] Yi H., Peng R., Zhang L.Y., Sun Y., Peng H.M., Liu H.D., Yu L.J., Li A.L., Zhang Y.J., Jiang W.H. (2017). LincRNA-Gm4419 knockdown ameliorates NF-kappaB/NLRP3 inflammasome-mediated inflammation in diabetic nephropathy. Cell Death Dis..

[84] Fu Y., Wang X., Zhang L., Ren Y., Hao L. (2022). Allograft inflammatory factor-1 enhances inflammation and oxidative stress via the NF-kappaB pathway in diabetic kidney disease. Biochem. Biophys. Res. Commun..

[85] Liu C., Yang M., Li L., Luo S., Yang J., Li C., Liu H., Sun L. (2022). A Glimpse of Inflammation and Anti-Inflammation Therapy in Diabetic Kidney Disease. Front. Physiol..

[86] Barutta F., Bruno G., Grimaldi S., Gruden G. (2015). Inflammation in diabetic nephropathy: Moving toward clinical biomarkers and targets for treatment. Endocrine.

[87] Sanchez-Nino M.D., Bozic M., Cordoba-Lanus E., Valcheva P., Gracia O., Ibarz M., Fernandez E., Navarro-Gonzalez J.F., Ortiz A., Valdivielso J.M. (2012). Beyond proteinuria: VDR activation reduces renal inflammation in experimental diabetic nephropathy. Am. J. Physiol. Renal. Physiol..

[88] Liu P., Li F., Xu X., Li S., Dong X., Chen L., Bai B., Wang Y., Qiu M., Dong Y. (2020). 1,25(OH)2D3 provides protection against diabetic kidney disease by downregulating the TLR4-MyD88-NF-kappaB pathway. Exp. Mol. Pathol..

[89] Afzal S., Bojesen S.E., Nordestgaard B.G. (2013). Low 25-hydroxyvitamin D and risk of type 2 diabetes: A prospective cohort study and metaanalysis. Clin. Chem..

[90] Fernández-Juárez G., Luño J., Barrio V., de Vinuesa S.G., Praga M., Goicoechea M., Lahera V., Casas L., Oliva J. (2013). 25 (OH) vitamin D levels and renal disease progression in patients with type 2 diabetic nephropathy and blockade of the renin-angiotensin system. Clin. J. Am. Soc. Nephrol..

[91] Liu Q., Sun J., Xu T., Bian G., Yang F. (2022). Associations of serum amyloid A and 25-hydroxyvitamin D with diabetic nephropathy: A cross-sectional study. J. Clin. Lab. Anal..

[92] Zhou T., Shen L., Li Z., Jia J., Xing H., Wang N., Jiao Q., Fan Y. (2022). Severe 25-Hydroxyvitamin D Deficiency May Predict Poor Renal Outcomes in Patients With Biopsy-Proven Diabetic Nephropathy. Front. Endocrinol..

[93] Abdel-Rahman E.M., Saadulla L., Reeves W.B., Awad A.S. (2012). Therapeutic modalities in diabetic nephropathy: Standard and emerging approaches. J. Gen. Intern. Med..

[94] Krairittichai U., Mahannopkul R., Bunnag S. (2012). An open label, randomized controlled study of oral calcitriol for the treatment of proteinuria in patients with diabetic kidney disease. J. Med. Assoc. Thail..

[95] Bonakdaran S., Hami M., Hatefi A. (2012). The effects of calcitriol on albuminuria in patients with type-2 diabetes mellitus. Saudi J. Kidney Dis. Transplant..

[96] Mao L., Ji F., Liu Y., Zhang W., Ma X. (2014). Calcitriol plays a protective role in diabetic nephropathy through anti-inflammatory effects. Int. J. Clin. Exp. Med..

[97] Thethi T.K., Bajwa M.A., Ghanim H., Jo C., Weir M., Goldfine A.B., Umpierrez G., Desouza C., Dandona P., Fang-Hollingsworth Y. (2015). Effect of paricalcitol on endothelial function and inflammation in type 2 diabetes and chronic kidney disease. J. Diabetes Complicat..

[98] Liyanage P., Lekamwasam S., Weerarathna T.P., Liyanage C. (2018). Effect of Vitamin D therapy on urinary albumin excretion, renal functions, and plasma renin among patients with diabetic nephropathy: A randomized, double-blind clinical trial. J. Postgrad. Med..

[99] Barzegari M., Sarbakhsh P., Mobasseri M., Noshad H., Esfandiari A., Khodadadi B., Gargari B.P. (2019). The effects of vitamin D supplementation on lipid profiles and oxidative indices among diabetic nephropathy patients with marginal vitamin D status. Diabetes Metab. Syndr..

[100] Liyanage G., Lekamwasam S., Weerarathna T., Liyanage C. (2021). Effect of vitamin D therapy on bone mineral density in patients with diabetic nephropathy; a randomized, double-blind placebo controlled clinical trial. J. Diabetes Metab. Disord..

[101] de Zeeuw D., Agarwal R., Amdahl M., Audhya P., Coyne D., Garimella T., Parving H.H., Pritchett Y., Remuzzi G., Ritz E. (2010). Selective vitamin D receptor activation with paricalcitol for reduction of albuminuria in patients with type 2 diabetes (VITAL study): A randomised controlled trial. Lancet.

[102] Kim M.J., Frankel A.H., Donaldson M., Darch S.J., Pusey C.D., Hill P.D., Mayr M., Tam F.W. (2011). Oral cholecalciferol decreases albuminuria and urinary TGF-β1 in patients with type 2 diabetic nephropathy on established renin-angiotensin-aldosterone system inhibition. Kidney Int..

[103] Huang Y., Yu H., Lu J., Guo K., Zhang L., Bao Y., Chen H., Jia W. (2012). Oral supplementation with cholecalciferol 800 IU ameliorates albuminuria in Chinese type 2 diabetic patients with nephropathy. PLoS ONE.

[104] Ahmadi N., Mortazavi M., Iraj B., Askari G. (2013). Whether vitamin D3 is effective in reducing proteinuria in type 2 diabetic patients?. J. Res. Med. Sci..

[105] Joergensen C., Tarnow L., Goetze J.P., Rossing P. (2015). Vitamin D analogue therapy, cardiovascular risk and kidney function in people with Type 1 diabetes mellitus and diabetic nephropathy: A randomized trial. Diabet. Med..

[106] Munisamy S., Daud K.M., Mokhtar S.S., Rasool A.H.G. (2016). Effects of 1α-Calcidol (Alfacalcidol) on Microvascular Endothelial Function, Arterial Stiffness, and Blood Pressure in Type II Diabetic Nephropathy Patients. Microcirculation.

[107] Tiryaki Ö., Usalan C., Sayiner Z.A. (2016). Vitamin D receptor activation with calcitriol for reducing urinary angiotensinogen in patients with type 2 diabetic chronic kidney disease. Ren. Fail..

[108] Peng Y., Li L.J. (2015). Serum 25-hydroxyvitamin D level and diabetic nephropathy in patients with type 2 diabetes mellitus. Int. Urol. Nephrol..

[109] Shao Y., Lv C., Yuan Q., Wang Q. (2016). Levels of Serum 25(OH)VD3, HIF-1α, VEGF, vWf, and IGF-1 and Their Correlation in Type 2 Diabetes Patients with Different Urine Albumin Creatinine Ratio. J. Diabetes Res..

[110] Navaneethan S.D., Schold J.D., Arrigain S., Jolly S.E., Jain A., Schreiber M.J., Simon J.F., Srinivas T.R., Nally J.V. (2011). Low 25-hydroxyvitamin D levels and mortality in non-dialysis-dependent CKD. Am. J. Kidney Dis..

[111] Xiao X., Wang Y., Hou Y., Han F., Ren J., Hu Z. (2016). Vitamin D deficiency and related risk factors in patients with diabetic nephropathy. J. Int. Med. Res..

[112] Ray S., Beatrice A.M., Ghosh A., Pramanik S., Bhattacharjee R., Ghosh S., Raychaudhury A., Mukhopadhyay S., Chowdhury S. (2017). Profile of chronic kidney disease related-mineral bone disorders in newly diagnosed advanced predialysis diabetic kidney disease patients: A hospital based cross-sectional study. Diabetes Metab. Syndr..

[113] Duan S., Lu F., Wu B., Zhang C., Nie G., Sun L., Huang Z., Guo H., Zhang B., Xing C. (2022). Association of Serum 25 (OH) Vitamin D With Chronic Kidney Disease Progression in Type 2 Diabetes. Front. Endocrinol..

[114] Liang Q., Hu H., Wu H., Chen X., Wang W., Le Y., Yang S., Jia L. (2021). A Nonlinear Relationship Between Serum 25-Hydroxyvitamin D and Urine Albumin to Creatinine Ratio in Type 2 Diabetes: A Cross-Sectional Study in China. Diabetes Metab. Syndr. Obes. Targets Ther..

[115] Xu F., Lu H., Lai T., Lin L., Chen Y. (2022). Association between Vitamin D Status and Mortality among Adults with Diabetic Kidney Disease. J. Diabetes Res..

[116] Vojtková J., Ciljaková M., Vojarová L., Janíková K., Michnová Z., Sagiová V. (2012). Hypovitaminosis D in children with type 1 diabetes mellitus and its influence on biochemical and densitometric parameters. Acta Med..

[117] Humalda J.K., Goldsmith D.J., Thadhani R., de Borst M.H. (2015). Vitamin D analogues to target residual proteinuria: Potential impact on cardiorenal outcomes. Nephrol. Dial. Transplant..

[118] Freundlich M., Gamba G., Rodriguez-Iturbe B. (2021). Fibroblast growth factor 23-Klotho and hypertension: Experimental and clinical mechanisms. Pediatr. Nephrol..

[119] Wu C.C., Liao M.T., Hsiao P.J., Lu C.L., Hsu Y.J., Lu K.C., Chu P. (2020). Antiproteinuria Effect of Calcitriol in Patients With Chronic Kidney Disease and Vitamin D Deficiency: A Randomized Controlled Study. J. Ren. Nutr..

[120] Bilezikian J.P., Formenti A.M., Adler R.A., Binkley N., Bouillon R., Lazaretti-Castro M., Marcocci C., Napoli N., Rizzoli R., Giustina A. (2021). Vitamin D: Dosing, levels, form, and route of administration: Does one approach fit all?. Rev. Endocr. Metab. Disord..

[121] Thadhani R., Appelbaum E., Pritchett Y., Chang Y., Wenger J., Tamez H., Bhan I., Agarwal R., Zoccali C., Wanner C. (2012). Vitamin D therapy and cardiac structure and function in patients with chronic kidney disease: The PRIMO randomized controlled trial. JAMA.

[122] Fakhoury M., Levy R., Melamed M.L. (2019). Vitamin D deficiency and kidney hyperfiltration: A mechanism of kidney injury?. Ann. Transl. Med..

[123] Krummel T., Ingwiller M., Keller N., Prinz E., Charlin E., Bazin D., Hannedouche T. (2022). Effects of high- vs low-dose native vitamin D on albuminuria and the renin-angiotensin-aldosterone system: A randomized pilot study. Int. Urol. Nephrol..

